# Assessing the connectivity value of roadway structures for terrestrial mammals across the Northern Appalachian forest of Vermont

**DOI:** 10.1371/journal.pone.0331493

**Published:** 2025-09-04

**Authors:** Caitlin E. Drasher, Chris Slesar, Jens Hawkins-Hilke, Glenn Gingras, Paul Marangelo, Vincent Landau, Kimberly R. Hall, Schuyler B. Pearman-Gillman, James D. Murdoch

**Affiliations:** 1 Wildlife and Fisheries Biology Program, Rubenstein School of Environment and Natural Resources, University of Vermont, Burlington, Vermont, United States of America; 2 Vermont Agency of Transportation, Barre, Vermont, United States of America; 3 Department of Fish and Wildlife, Vermont Agency of Natural Resources, Essex, Vermont, United States of America; 4 The Nature Conservancy Vermont, Montpelier, Vermont, United States of America; 5 Conservation Science Partners, Inc., Truckee, California, United States of America; 6 Vibrant Planet PBC, Incline Village, NV, United States of America; 7 North America Science, The Nature Conservancy, Haslett, Michigan, United States of America; 8 Apex Resource Management Solutions Ltd, Ottawa, Ontario, Canada; Instituto Federal de Educacao Ciencia e Tecnologia Goiano - Campus Urutai, BRAZIL

## Abstract

Landscape connectivity is often negatively impacted by road networks that fragment habitat and result in genetic and demographic consequences for wildlife. Existing roadway structures like bridges, culverts, and underpasses can facilitate connectivity and reduce the barrier effect of roads by providing less risky areas for animals to cross. Estimating areas of high wildlife movement near roads is beneficial for prioritizing transportation investments for wildlife. We used an omnidirectional circuit theory approach to model the movements of eight terrestrial mammal species across the state of Vermont, a forested region central to the globally important Northern Appalachian ecoregion. We combined expert-derived landscape resistance surfaces with wildlife occurrence data to develop species-specific connectivity models at statewide (23,873 km^2^, 30 m resolution) and roadway structure (100 m radius around 5,912 structures, 0.5 m resolution) scales. The flow of animal movement across the landscape, depicted as electrical current density, was highest for forest-obligate species along the forested, mid-elevation foothills of the Green Mountains in central Vermont and lowest in the agricultural Champlain Valley; however, for more urban- and agriculture-adapted species, flow was highest in developed areas and lower elevation valleys. Average current density was highest for black bear (*Ursus americanus*), and lowest for striped skunk (*Mephitis mephitis*) at the statewide scale and highest for raccoon (*Procyon lotor*) and lowest for moose (*Alces alces*) at the finer structure scale. Results at both scales revealed different patterns of expected animal movement that reflect the relative extent of connectivity. We then scored connectivity for each structure across all species by combining both scales using four different methods to capture a range of management interests. Rankings varied greatly depending on the method used, highlighting the need to clearly articulate objectives when scoring structures or other features in a landscape. Resistance, occupancy, and current maps also indicated the broad importance of intact forest for connectivity and may be particularly important for identifying priority regions for protection under Vermont’s Community Resilience and Biodiversity Protection Act that mandates protecting 50% of the state by 2050.

## Introduction

Wildlife populations often depend on landscape connectivity for persistence [1]. A connected landscape provides access to resources, opportunities for dispersal, and allows for genetic exchange between subpopulations [2,3]. Landscape fragmentation can lead to changes in distribution and create isolated populations, which often results in negative impacts to species [1–4]. Globally, terrestrial mammals experiencing higher levels of fragmentation are consequently at a greater risk of extinction [5].

Road networks are wide-reaching and contribute to landscape fragmentation [6,7]. In the United States (US), there are 6.7 million km of public roads that are traveled over 5 trillion km each year [8]. While road networks provide connectivity for human society, they generally decrease connectivity for wildlife through subdivision of habitat [9,10]. Roads cause direct mortality to wildlife through vehicle collisions, with incidents involving large mammals costing US motorists nearly $8.4 billion annually [11]. Roads cause indirect mortality to wildlife by inhibiting gene flow, potentially resulting in less viable populations [12–14] and evolutionary consequences in some cases [15]. Impacts from roadways also vary by species and taxa, and these impacts sometimes extend beyond the actual road footprint [6]. Understanding and mitigating the effects of roads is especially important in the face of climate change, as species are expected to experience range shifts in response to changes in their natural environment [3,16].

Several approaches have been used to mitigate the negative effects of roads on wildlife. Properly designed roadway structures can facilitate wildlife movement across road networks, increasing the permeability of roads [10,17]. Culverts, bridges, underpasses, and overpasses provide opportunities for wildlife to navigate complex transportation networks and reduce the risk of collision [10,18]. Existing structures like culverts are often abundant along roadways; however, some structures are better equipped to facilitate wildlife passage than others [19]. The physical attributes of roadway structures, such as width, length, substrate, and material, may promote or deter wildlife use, depending on species size and movement preferences [17,20–23]. Transportation and wildlife managers can improve existing structures to accommodate a wider range of species [24], yet these projects are often very costly, and it is beneficial to prioritize investments in wildlife-focused infrastructure improvements to areas of the landscape and road network that maximize wildlife connectivity [23,25–27].

To locate areas along roads where investments will be most impactful for improving landscape connectivity, factors considered may include the broader landscape context influencing species movements, as well as the immediate landcover features surrounding existing roadway structure locations [28]. Wildlife movement and landscape connectivity are often scale-dependent [29]. At the landscape scale, wildlife movements become channeled through specific areas based on landcover composition and configuration, and structures that are located within migration paths, corridors, or pinch-points often experience higher use [21,23]. At a finer scale, the presence of and distance to vegetative cover in the immediate vicinity of a structure can influence use [21,24]. Thorough mitigation planning considers multiple spatial scales when prioritizing structure improvements to maximize the probability of use by wildlife species [28]. For instance, a culvert or bridge located outside of known wildlife corridors in the landscape may not experience high levels of use, even if improvements to the immediate site-level landcover and structure characteristics are implemented [21].

Previous studies have used connectivity modeling to identify locations where species are more likely to cross the road to guide the prioritization of mitigation investments [25,27]. For example, Zeller et al. [27] compared multiple connectivity models to identify road crossing hotspots for black bears (*Ursus americanus*) in Massachusetts, USA including resource selection functions, least-cost paths, resistant kernels, individual based movement models, and a circuit-theory approach using Circuitscape [30,31]. The circuit-theory approach outperformed all other techniques and has been widely applied to connectivity modeling elsewhere [27,32]. This approach models species movements as the flow of electricity through a circuit [30] and incorporates information on the resistance of landscape features, including roads, to the movement of a given species [30,31,33]. Areas of concentrated electricity flow indicate increased predicted wildlife movement, which can inform conservation planning and decision-making [33]. In the context of roads, areas of concentrated flow near existing roadway structures may suggest locations where mitigation activities would be most beneficial.

Landscape resistance surfaces have been widely used in ecological and circuit-based modeling to predict the movement of animals across landscapes, and species-specific resistance surfaces are more precise in explaining wildlife connectivity than generalized approaches that group multiple species into a single model of landscape resistance [34,35]. Species responses to landscape variables differ based on their ecological needs and movement behaviors [35]. For example, human development variables (e.g., roads, buildings) generally present a high level of resistance to the movements of many forest obligate species; however, these same areas may be of low resistance to more generalist species that are adapted to anthropogenic environments. Additionally, the spatial resolution (or pixel size) of resistance surfaces is important for landscape connectivity analyses as some species may respond to fine-scale landscape features, while the movement of other species may be explained by coarser patterns of landscape composition [34,35].

A useful innovation in circuit theory modeling is the development of the Omniscape approach [33,36]. Omniscape allows electricity to move in all directions (or omnidirectionally) throughout the landscape using a moving window technique and incorporates a ‘source-strength’ input that represents the abundance of a species across the analysis area, providing a starting point for electricity that then moves through the resistance surface. As spatial models of abundance are often challenging to develop, maps of distribution based on occupancy or habitat suitability can serve as a proxy for abundance [36]; Pearman-Gillman et al., *in review*]. Omnidirectional connectivity models reduce biases associated with manually choosing starting and ending points of electricity and are useful when source locations are unknown or when modeling connectivity for multiple species regionally [37]. Omnidirectional landscape connectivity models may also be ideal for species that do not have more predictable migratory patterns between fixed starting and ending locations in a landscape; for example, some western ungulate species exhibit large-scale migrations between seasonal resources [38], whereas mammals in the eastern US maintain home ranges and do not exhibit such large-scale migrations.

We focused on the US state of Vermont to create multi-scale and species-specific omnidirectional connectivity models for terrestrial mammal species, and quantify connectivity around roads and existing roadway structures (culverts, bridges, underpasses) to guide transportation infrastructure mitigation investments. Vermont wildlife and transportation agencies have identified mitigating the impact of roads on wildlife as a goal in their long-term management plans [39–41]. To help prioritize wildlife-focused transportation infrastructure investments in Vermont, transportation managers identified the need to predict areas of higher probable movement of terrestrial species across the road network. By ranking existing roadway structures by their connectivity value for terrestrial mammal species, wildlife improvements can be implemented at high-priority structures as opportunities arise. Omnidirectional models of wildlife movement can be used to quantify and rank the relative value of roadway structures for maintaining landscape connectivity throughout the state to inform transportation management actions that may improve connectivity in high-movement areas.

The goal of our study was to model wildlife movement in Vermont to assess the connectivity value of existing structures along the road network. Our objectives were to 1) create landscape resistance surfaces for eight terrestrial mammal species based on ecologically relevant landcover variables, 2) model the movement of these species in Vermont at two spatial scales, the landscape scale (statewide) and roadway structure scale (individual structure locations), and 3) quantify connectivity at structure locations and combine multi-scale results in several ways to rank individual roadway structures by their collective connectivity value across focal species. The multi-scale and multi-species assessment of structure connectivity values provides important information for decision-making to improve connectivity across road networks.

## Methods

### Study area

Vermont is centrally located within the Northern Appalachian/Acadian Ecoregion, which covers 330,000 km^2^ from New York through New Hampshire, Maine, and the Canadian province of Québec [12]. The Appalachians are considered a globally significant region for biodiversity and part of The Nature Conservancy’s Resilient and Connected Network of lands identified as critically important for tackling climate change and conserving biodiversity [42]. The central location of Vermont in the northern Appalachians makes it a key connector that facilitates species movements regionally to other states and provinces. Vermont contains portions of 6 of 10 connectivity corridors for wildlife identified by the Staying Connected Initiative – a consortium of federal, state, non-profit, private, and academic partners [43]. Moreover, the Green Mountain range in Vermont has been identified as a priority area for connectivity within the broader ecoregion and is vulnerable to future human development [12]. Vermont is also located in a unique area of high to very high climate flow and predicted to be an influential connector facilitating the movement of Appalachian species northward as climate changes [42].

**Fig 1:**
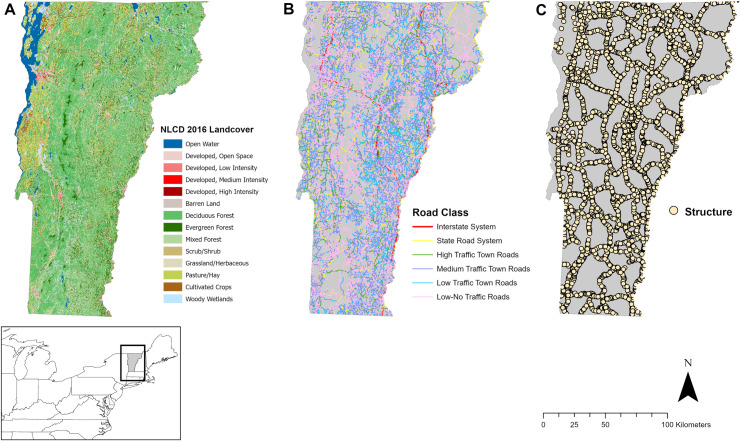
Landcover data (a) [44] and roadways classified into six general categories (b) in Vermont, USA. We focused our analysis on 5,912 state-managed transportation structure locations on the Interstate System and State Road System (c).

Vermont is a rural state covering 23,873 km^2^ with approximately 645,000 residents (Fig 1) [45]. It is 78% forested, consisting primarily of northern hardwood forest and spruce-fir forest types [46]. Elevation ranges from 29 m along the shores of Lake Champlain to 1,339 m in alpine zones of the Green Mountains [46]. Development is more concentrated in the lowland riparian basins including the Champlain and Connecticut River valleys and along riparian corridors [46]. Agriculture is prominent in the Champlain Valley, and residential development has increased significantly in recent decades, expanding faster than the state’s population growth [46]. Vermont is bordered by the US states of New York, Massachusetts, and New Hampshire, and shares a northern border with the Canadian province of Québec (Fig 1).

The Vermont road network includes 11,508 km of paved roads and 13,920 km of unpaved roads, totaling 25,428 km of roadway [47] (Fig 1A). This includes larger interstate highways (I-89, I-91, I-93), multiple state highways, and other class I-IV roads managed by the state and local municipalities (Fig 1B). The public drives an estimated 11.8 billion km annually on this road network [48]. The Vermont Agency of Transportation maintains over 50,000 roadway structures (bridges, arch/box/pipe culverts, other underpasses) of varying size and condition, on state roads and some town roads. Many of these structures facilitate the movement of water beneath the roadway, either for intermittent drainage or to pass traffic over rivers and streams. Road classes were grouped into six categories based on size and traffic volume, with input from the Vermont Agency of Transportation, for use in resistance-based connectivity modeling. We selected 5,912 state-managed roadway structures that were >1 m in diameter for analysis (Fig 1C) [49–53]. Structures above this threshold were identified as priority structures by the transportation agency since many were already large enough to facilitate movement for many terrestrial mammal species or may be more easily retrofitted to better accommodate species movements.

### Focal species

We focused on eight terrestrial mammal species: black bear (*Ursus americanus*), bobcat (*Lynx rufus*), coyote (*Canis latrans*), moose (*Alces alces*), raccoon (*Procyon lotor*), red fox (*Vulpes vulpes*), striped skunk (*Mephitis mephitis*), and white-tailed deer (*Odocoileus virginianus*). These species hold ecological, economic, and cultural significance in Vermont and the broader region, and are wide-ranging and frequently encounter roadways [22,54]. These species are also target species for wildlife management by the transportation and wildlife agencies in the state and selected with input from agency partners [39,41]. The selected species are frequently involved in road collisions, Species of Greatest Conservation Need, high value species for hunting and other forms of recreation, or priority species for the US Department of Agriculture Rabies Management Program [55]. Predicting the movements of these species across the state may have broader implications for species and land management, wildlife disease monitoring, and road collision mitigation.

### Objective 1: Create landscape resistance surfaces

We generated species-specific resistance surfaces at two spatial resolutions using an expert elicitation process (methods reviewed and IRB exemption was determined by the University of Vermont Internal Review Board). We conducted an online survey of wildlife experts that asked them to score landscape resistance for land cover variables for each species following Dickson et al. [56]. We invited 10 regional wildlife professionals with expertise in one or more of the focal species to complete species-specific surveys (1 to 3 experts per species). Experts selected were those actively working with one or more of the focal species professionally as state or federal wildlife biologists and managers in Vermont and the broader New England region. All experts were recruited to participate in the survey between April 1 and August 31, 2021, and viewed a pre-survey video that explained the project background, described resistance surfaces and their use in Omniscape connectivity modeling, and walked through the resistance surface survey process. Experts were also provided with a written project information sheet with the survey and informed that any participation in the written survey implied consent. We developed the survey using the LimeSurvey platform (LimeSurvey GmbH 2005; S1 Fig).

Experts scored resistance values of landcover variables in two datasets for use in each spatial scale of the wildlife movement analysis: 1) Landscape scale: 2016 National Landcover Dataset (NLCD) variables (30 m resolution) [44], and 2) Structure scale: 2016 Vermont Center for Geographic Information (VCGI) lidar dataset variables (0.5 m resolution) [57]. In addition to the fifteen variables scored in the NLCD dataset for the landscape scale analyses, we included the six categories of road classes for scoring, and these road class variables were overlaid onto the NLCD raster after removing road pixels that were classified generally as development in NLCD. For the structure scale VCGI dataset, seven of the variables from the base landcover raster dataset were included for scoring, and the six road class categories were also overlaid onto this raster in place of the generic ‘road’ variable. Additionally, two supplemental VCGI vector datasets for agriculture and wetlands were converted to rasters and added to the VCGI raster dataset. In total, experts scored 21 variables in the landscape scale NLCD dataset and 15 variables in the structure scale VCGI dataset (Table 1).

**Table 1:** Landcover and road class variables used to develop landscape scale and structure scale resistance layers for 8 wildlife species in Vermont, USA. Landcover in the buffered analysis area within Québec, Canada used variables from the North American Land Change Monitoring System (NALCMS) dataset, and these variables were reclassified as comparable US National Land Cover Dataset (NLCD) variables; attribute names from original datasets shown.

Scale and variable name	Attribute name	Source
***Landscape scale***		
Open water	NLCD 11; NALCMS 18	Dewitz 2019; Canada Centre for Remote Sensing (CCRS) et al. 2020
Developed open space	NLCD 21	Dewitz 2019
Developed low intensity	NLCD 22	Dewitz 2019
Developed medium intensity	NLCD 23; NALCMS 17	Dewitz 2019; CCRS et al. 2020
Developed high intensity	NLCD 24	Dewitz 2019
Barren land	NLCD 31; NALCMS 16	Dewitz 2019; CCRS et al. 2020
Deciduous forest	NLCD 41; NALCMS 4, 5	Dewitz 2019; CCRS et al. 2020
Evergreen forest	NLCD 42; NALCMS 1, 2, 3	Dewitz 2019; CCRS et al. 2020
Mixed forest	NLCD 43; NALCMS 6	Dewitz 2019; CCRS et al. 2020
Shrub/scrub	NLCD 52; NALCMS 7, 8, 11	Dewitz 2019; CCRS et al. 2020
Grassland/herbaceous	NLCD 71; NALCMS 9, 10, 12	Dewitz 2019; CCRS et al. 2020
Pasture/hay	NLCD 81; NALCMS 15	Dewitz 2019; CCRS et al. 2020
Cultivated crops	NLCD 82	Dewitz 2019
Woody wetlands	NLCD 90	Dewitz 2019
Emergent herbaceous wetlands	NLCD 95; NALCMS 95	Dewitz 2019; CCRS et al. 2020
***Landscape and structure scale***		
Interstate system	VTrans AOTCLASS>=50 and AOTCLASS<60 NHDOT Tier = 1 NYSDOT ACC in 1, 2 MassDOT F_class = 1	Vermont Agency of Transportation (VTrans) 2019, New Hampshire Department of Transportation (NHDOT) 2020, New York State Department of Transportation (NYSDOT) 2020, MassDOT 2020
State road system	VTrans AOTCLASS>=30 and AOTCLASS<50 NHDOT Tier = 2 NYSDOT ACC = 3 MassDOT F_class = 2	VTrans 2019, NHDOT 2020, NYSDOT 2020, MassDOT 2020
High traffic town	VTrans AOTCLASS in 1, 2 NHDOT Tier = 3 NYSDOT ACC 4 MassDOT F_class in 3,5	VTrans 2019, NHDOT 2020, NYSDOT 2020, MassDOT 2020
Medium traffic town	VTrans AOTCLASS = 3 NHDOT Tier = 4 NYSDOT ACC = 5 MassDOT F_class = 6	VTrans 2019, NHDOT 2020, NYSDOT 2020, MassDOT 2020
Low traffic town	VTrans AOTCLASS = 4 NHDOT Tier = 5 NYSDOT ACC = 6 MassDOT F_class = 0	VTrans 2019, NHDOT 2020, NYSDOT 2020, MassDOT 2020
Low/no traffic town	VTrans AOTCLASS in 5, 6, 7, 8, 9, 96, 97 NHDOT Tier = 6 NYSDOT FCC in A5, A7	VTrans 2019, NHDOT 2020, NYSDOT 2020,
***Structure scale***		
Canopy cover	VT Base Land Cover, 1	VCGI 2019
Grass/shrub	VT Base Land Cover, 2	VCGI 2019
Bare soil	VT Base Land Cover, 3	VCGI 2019
Water	VT Base Land Cover, 4	VCGI 2019
Building	VT Base Land Cover, 5	VCGI 2019
Other paved	VT Base Land Cover, 7	VCGI 2019
Railroad	VT Base Land Cover, 8	VCGI 2019
Agriculture	VT Agriculture Land Cover, 1–3	VCGI 2019
Wetland	VT Wetlands Land Cover, 1–3	VCGI 2019

Resistance scoring was based on a 1–100 scale, with 1 representing the lowest resistance to movement, 100 representing the highest resistance to movement, and NA (designated as 101 by the experts) representing a complete (impermeable) barrier. When multiple experts provided input for a species, resistance values for each variable were averaged together and used to create hypothesized resistance maps at each scale and then used in preliminary Omniscape analyses (see Objective 2 below). We produced draft Omniscape maps of statewide movement for each species at the landscape scale, and draft Omniscape maps of fine-scale movements for each species around 5 test structures at the structure scale.

Following these preliminary analyses, we conducted individual follow up meetings with each expert. Experts were presented with the draft Omniscape maps for their species at each scale and given the opportunity to ask interpretation questions and provide feedback on the preliminary results. Following the interview, experts were given one opportunity to adjust their initial resistance values to improve the accuracy of the maps if needed. Once again, resistance values (including any adjusted resistance values) were averaged together for each species and used to create the final resistance inputs used in the Omniscape analyses at each scale (Table 2).

**Table 2:** Resistance values for landscape variables in the landscape scale and structure scale analyses by species. Expert opinion values for each species were elicited through an online survey and follow-up interview with expert scores for each variable averaged together. Scores ranged from 1 indicating no resistance to 100 indicating complete resistance and ‘null’ for impermeable.

Variable	Black bear	Bobcat	Coyote	Moose	Raccoon	Red fox	Striped skunk	White-tailed deer
***Landscape scale analysis***								
Open water	77	83	65	35	53	60	73	95
Developed open space	67	70	30	58	6	35	1	50
Developed low intensity	67	70	30	58	6	35	1	50
Developed medium intensity	67	70	30	58	6	35	1	50
Developed high intensity	85	98	65	74	38	65	40	95
Barren land	60	43	35	49	78	25	15	50
Deciduous forest	1	2	1	1	1	6	4	1
Evergreen forest	1	3	1	1	1	6	3	1
Mixed forest	1	2	1	1	15	6	3	1
Shrub/scrub	14	1	6	1	30	1	6	1
Grasslands/herbaceous	48	70	19	27	10	3	1	5
Pasture/hay	48	70	19	27	10	3	1	5
Cultivated crops	40	72	30	39	3	5	6	20
Woody wetlands	3	1	1	1	8	3	11	5
Emergent herbaceous wetlands	20	18	8	17	8	5	15	85
High-traffic town roads	53	80	43	58	70	38	61	50
Moderate-traffic town roads	33	78	38	40	50	37	40	30
Low-traffic town roads	12	50	32	17	15	11	33	5
Low/no traffic roads	1	6	1	4	8	1	3	5
State road system	63	85	65	62	53	50	66	50
Interstate system	82	95	80	68	65	68	68	65
***Structure scale analysis***								
Buildings	Null	Null	Null	Null	Null	Null	Null	Null
Canopy cover	1	1	1	1	1	1	6	1
Grass/shrub	35	12	11	4	13	1	1	1
Agriculture	45	65	12	20	3	5	5	10
Wetland	8	3	5	7	10	3	10	20
Bare soil	35	68	45	7	20	15	28	5
Water	38	53	30	26	5	48	50	95
Other paved	58	83	63	42	8	45	28	80
Railroad	32	10	6	20	8	8	12	80
High-traffic town roads	68	73	58	65	50	25	65	90
Moderate-traffic town roads	47	43	15	58	50	8	65	70
Low-traffic town roads	20	20	14	19	10	6	30	5
Low/no traffic roads	5	3	1	2	1	1	3	5
State road system	73	83	75	68	75	58	65	90
Interstate system	90	95	90	75	75	68	65	90

### Objective 2: Model and map landscape connectivity

We used Omniscape [33,36] to model and map connectivity for each focal species across Vermont. Omniscape is a package run through the Julia programming language [36,58]. We modeled species-specific landscape connectivity with Omniscape at two spatial scales. At the landscape scale, we modeled connectivity across the entire state of Vermont at a 30 m resolution. At the structure scale, we modeled connectivity within a 100-m radius around each individual structure at a 0.5 m resolution; this radius and resolution is relevant for transportation management actions within the right-of-way of structures.

#### Moving window algorithm.

Omniscape uses a moving window algorithm to determine the extent to which electrical current can travel from any source location to ground locations in the landscape, and the size of the window may be specified to constrain the flow of electricity to a biologically reasonable distance [36]. Omniscape centers the moving window on a single center pixel in the landscape, clips the source and resistance layers to the designated moving window size, and all source pixels within the window will emit electricity that then travels through the resistance surface to reach the center (ground) pixel. This process is repeated across the landscape iteratively so that each pixel may act as a ground, and all moving window iterations are summed together to create a single map of cumulative current flow across the study area. The size of this moving window ultimately determines how far electricity may travel from each source pixel identified within the moving window back to the center ground pixel [33,36]. To represent general species movement capabilities, we set the moving window size to the average home range size of males and females for each species (Table 3).

**Table 3:** Average home range size of each focal species used to determine the moving window size in landscape scale Omniscape analyses.

Species	Mean home range for adults (km^2^)	Moving window radius (m)	Study location	Home range method	Reference
Black bear	80.22	5053.20	Vermont, USA	95% Kernel	Hammond (2002)
Bobcat	46.90	3863.77	Vermont, USA	Kernel	Donovan et al. (2011)
Coyote	17.90	2387.00	Vermont, USA	Harmonic mean, outliers removed	Person & Hirth (1991)
Moose	75.78	4911.37	Vermont, USA	95% Fixed-kernel	Blouin et al. (2021)
Raccoon	0.83	514.00	Ontario, Canada	95% Fixed-kernel	Rosatte et al. (2010)
Red fox	14.70	2163.14	Maine, USA	Convex polygon, outliers removed	Harrison et al. (1989)
Striped skunk	0.90	535.24	Ontario, Canada	100% MCP	Rosatte et al. (2011)
White-tailed deer	11.42	1906.59	Québec, Canada	95% MCP	Lesage et al. (2000)

### Source-strength and resistance inputs

The source-strength input for the analysis determines where electricity is emitted from in the landscape, and how much electricity is emitted from each pixel in a raster map [36]. The source-strength value within pixels ranges from 0 to 1, where pixels with a value closer to 0 emit less electricity, and those with values closer to 1 emit more electricity, corresponding with the predicted amount of individuals coming out of each location in the landscape [36]. We used regional species-specific occupancy models as the source-strength input for connectivity analyses at both the landscape and structure scales (S2 Table) [59]. These occupancy models were generalized linear mixed effects models developed with a model selection approach that analyzed the effects of 74 variables across the New England landscape, and each model performed well against independent empirical wildlife data [59]. These 30 m resolution occupancy models were used at the same 30 m resolution for use in the landscape scale connectivity models, and resampled to 0.5 m resolution for use in the structure scale models.

Once electrical current is emitted from a source pixel it travels toward a ground pixel, and the resistance input determines the path and strength of electricity flow based on the relative cost of movement through each pixel [33,60]. The final species-specific resistance surfaces created in Objective 1 were used as resistance inputs for species-specific Omniscape analyses at each scale (Table 2).

### Analysis area

Both the landscape scale and structure scale analyses focused within the bounds of Vermont. However, to avoid edge effects at the landscape scale, the resistance inputs were buffered by 10 km (a distance greater than the moving window radii for each species) into the neighboring New York, Massachusetts, and New Hampshire. Resistance inputs were also buffered into Québec; however, due to differences in landcover data, the North American Land Change Monitoring System (NALCMS) landcover dataset [61] was used over the northern border (similar categories of NALCMS landcover were grouped to coincide with NLCD landcover variables as needed; Table 1). Source-strength inputs were also buffered into the neighboring states and province; however, occupancy data were unavailable in New York and Québec, and therefore approximate source-strength values were inferred in these areas using a Focal Statistics averaging process in ArcGIS (v. 10.8.1, ESRI, Redlands, California, USA) for each species-specific occupancy input (S3 Fig). Buffering the resistance and source-strength inputs allowed Omniscape to start the analysis outside of Vermont, while results were clipped to the boundary of Vermont. Buffering allowed electricity to flow into and out of Vermont, preventing pooling of electricity at the borders which can result in artificially high current densities in border areas [33,62].

At the structure scale, we used a Vermont-specific high-resolution (0.5 meter) landcover dataset for the resistance input, and therefore could not buffer into neighboring states or province to avoid edge effects. However, due to the small Omniscape analysis area around individual structures (100 m) at this scale, less than 1% of structures had radii that extended over the Vermont border (n = 47). Structures located within this edge effect zone may experience some edge effects.

### Computation

Landscape scale analyses were run with Omniscape.jl version 0.5.0 (Julia version 1.5.3) [36,58]. Structure scale analyses were run using the VermontTerrestrialPassageTool.jl, a Julia package developed for this analysis to cycle through all structure locations and run targeted Omniscape.jl analyses within a 100-m radius of each structure [63]. All analyses were run on the Bluemoon cluster, a 161-node computing cluster with 8392 compute cores with up to 4 TB RAM, of the University of Vermont Advanced Computing Center (Burlington, Vermont, USA). Landscape scale analyses ran on one node and averaged 3.7 hours to complete per species (maximum compute time of 13.8 hours), and structure scale analyses ran on one node and averaged 84.3 hours to complete per species (maximum compute time 85.7 hours).

### Objective 3: Rank roadway structures

We quantified connectivity around each existing roadway structure location from each species-specific connectivity map for each spatial scale. We then combined species-specific results to rank each structure by overall connectivity value for all species. First, we calculated general current density (CD) metrics for each individual structure location: 1) CD_meanLS_ representing the sum of species-specific mean current density values within 1 km of each structure from each landscape scale (LS) map; and 2) CD_meanSS_ representing the sum of species-specific mean current density values within 50 m of each structure from each structure scale (SS) analysis. This resulted in 5,912 CD_meanLS_ values (sum of species-specific means at each location from LS results) and 5,912 CD_meanSS_ values (sum of species-specific means at each location from SS results).

We then used four different methods of combining connectivity metrics from each spatial scale into a single ranking for each structure, to rank structures from highest overall connectivity value to lowest for all species combined. We used Spearman’s rank correlation to explore relationships between the rankings generated by each method [64].

- Method 1: Landscape Scale Mean Current Density. This method assessed the overall connectivity value of roadway structures based on the average electrical current density surrounding them from the landscape scale analysis. For each structure, the CD_meanLS_ was calculated, and all 5,912 structures were ranked from highest CD_meanLS_ to lowest. Structures with a higher CD_meanLS_ rank have more electrical current surrounding them within a 1 km radius, representing higher priority connectivity locations. This method aimed to quantify average predicted wildlife connectivity around roadway structures based on the broader movement patterns of the species statewide.
- Method 2: Structure Scale Mean Current Density. This method assessed the value of roadway structures based on the average electrical current density surrounding them from the structure scale analysis. For each structure, the CD_meanSS_ was calculated, and all 5,912 structures were ranked from highest CD_meanSS_ to lowest. Structures with a higher CD_meanSS_ rank had more electrical current surrounding them within a 50-m radius and represent higher priority wildlife movement locations based on finer-scale species movements modeled at a 0.5 m resolution only in the immediate vicinity of structures. This method aimed to quantify average predicted wildlife movement around roadway structure locations based on the immediate composition and configuration of landcover at a finer resolution around structures.
- Method 3: Sum of Landscape Scale and Structure Scale Mean Current Densities. This method assessed the overall connectivity value of roadway structures by summing the average electrical current density surrounding them in each scale of analysis. For each structure, the landscape scale CD_meanLS_ and the structure scale CD_meanSS_ were summed to get a multi-scale measure of mean current density (CD_meanLS+meanSS_). Structures were ranked from highest to lowest CD_meanLS+meanSS_. This rank considered both the broad statewide species movements as well as fine-scale movements around structures.
• Method 4: Landscape Scale Rarity-Weighted Richness Index. This method accounted for any rarity of species-specific current flow around structures, and ranked structures that may have rare/less flow for one or more species higher compared to other structures. We calculated a modified Rarity-Weighted Richness Index (RWRI) [65] for each structure from species-specific mean current density values within 1 km of each structure from the landscape scale maps. This method evaluated structures based on whether they contained rare species flow around them compared to all other ranked structures. For each site, RWRI was calculated as:

$$\sum_{\mathrm{\backslash emph}i=1}^{\mathrm{\backslash emph}n}\left(1/h_{\mathrm{\backslash emph}i}\right)$$

Where h_i_ is the number of sites containing electrical flow for species i, and n representing the total number of species with flow at a given site.

### Evaluation of connectivity models

We used an independent set of wildlife data collected by state transportation and wildlife agencies to evaluate roadway structure connectivity scores at each spatial scale [66]. Camera trap data were collected from 2015 to 2021 at 49 roadway structure locations across the state that were predicted to be ideal for wildlife movement based on the expert opinion of wildlife and transportation managers [22]. We calculated the probability of species passing through a structure across camera location in two categories: 1) ungulates (moose, deer), and 2) carnivores (bear, bobcat, coyote, red fox, skunk; raccoon detections were not recorded in this dataset). We then compared these estimates of passage to the landscape scale and structure scale ‘connectivity scores’ at each structure. We assumed that likelihood of passage through structures would correlate to connectivity scores. As sites were considered ‘ideal’ for crossing, we expected passage rates and connectivity scores to be high and roughly equivalent if connectivity results were accurate. Probability and connectivity values were standardized on a scale from 1–100 to make comparisons.

## Results

### Objective 1: Landscape resistance surfaces

All 10 regional wildlife experts responded to the survey, with some experts (n = 5) completing the survey for multiple species given their expertise. For each species, we used input from 1–3 experts to generate resistance values for landscape variables at both spatial scales. Statewide mean resistance was highest for bobcat (19.33 ± 30.53 SE), and lowest for skunk (8.40 ± 16.30 SE). For other medium-sized mammals, statewide mean resistance was 8.78 ± 16.23 for coyote, 9.10 ± 14.82 for raccoon, and 9.62 ± 13.24 for red fox. For larger mammals, statewide mean resistance was 13.68 ± 23.11 for bear, 9.41 ± 15.87 for moose, and 9.03 ± 21.62 for white-tailed deer. Based on the composition of landcover variables in the Vermont landscape, most species had a mean landscape resistance of 9 on a 1 to 101 scale across the study area. Final landscape resistance values (Table 2), determined by an average across expert values, were used as an input in the Omniscape analyses.

### Objective 2: Wildlife connectivity models

**Fig 2:**
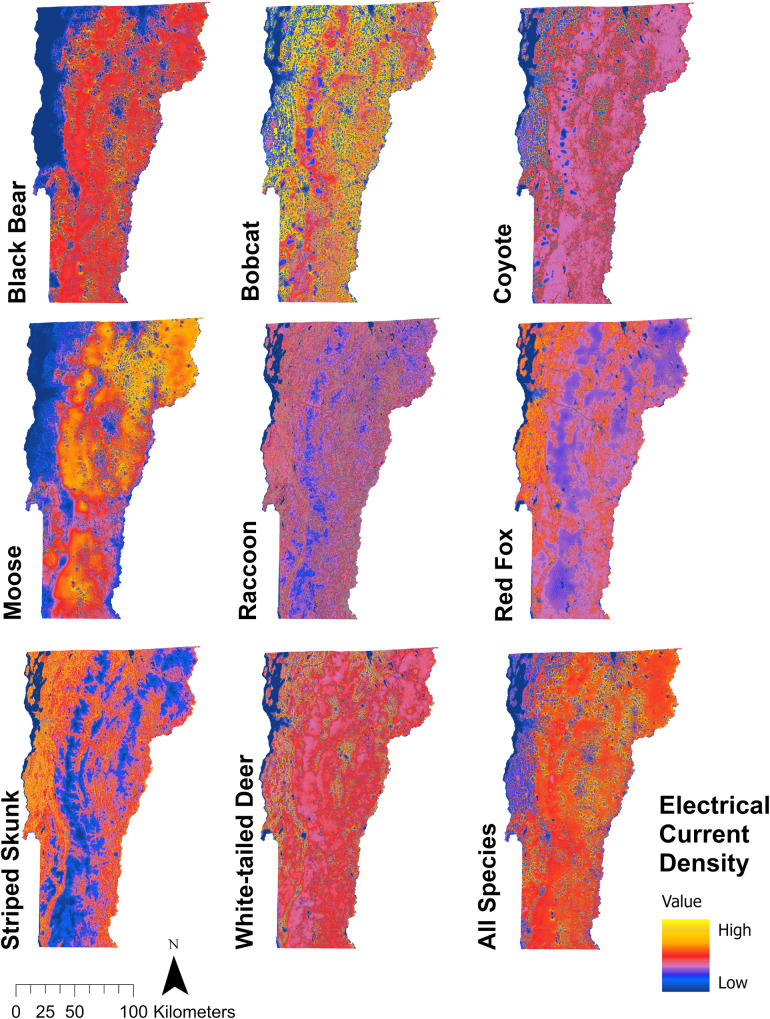
Maps of predicted wildlife movement throughout Vermont from Omniscape analyses. Areas of high electrical current density represent areas of more concentrated expected species movement.

We developed 8 species-specific maps showing statewide landscape connectivity at the landscape scale and summed these maps together to visualize statewide connectivity patterns of all species combined (Fig 2). From this combined map, statewide mean electrical current density was 388.35 ± 147.82 SE amps for all species combined. Mean electrical current density was highest for bear (94.91 ± 53.76 SE) and lowest for skunk (8.39 ± 4.67 SE) (Table 4). Flow was generally higher along the spine of the Green Mountains and in areas of lower human population density for more forest-obligate species, such as bear and moose. Bobcat, coyote, and white-tailed deer had intermediate levels of flow statewide, with pinch points of concentrated flow in agricultural areas where electrical current became more constrained by roads and cropland. The more urban- and agricultural-adapted species, raccoon, red fox, and skunk, had highest flow in lower elevation areas (such as the Champlain and Connecticut River valleys) and along corridors of human development (including the US Route-7 corridor and the greater Burlington and Montpelier areas).

**Table 4:** Electrical current density summary statistics (in amps of electricity) for each species at the landscape scale (calculated from the statewide maps within 1 km radius of all structures), and among all transportation structure locations (calculated within the 50 m radius of all structures) at the structure scale.

Species	Min	Max	Mean	SD
***Landscape scale***				
All species combined*	0.40	3255.55	388.35	147.8
Black bear	0.02	1628.99	94.91	53.76
Bobcat	0.47	1242.80	69.66	35.11
Coyote	0.00	552.48	56.10	24.22
Moose	0.00	952.44	71.10	40.94
Raccoon	0.00	72.43	11.70	4.92
Red fox	0.00	312.29	34.09	12.25
Striped skunk	0.00	60.278	8.39	4.67
White-tailed deer	0.00	402.85	42.65	15.72
***Structure scale***				
All species combined*	0.00	14454.30	361.55	74.12
Black bear	0.00	3284.86	81.13	44.84
Bobcat	0.00	2397.57	84.55	25.92
Coyote	0.00	1969.84	115.56	26.53
Moose	0.00	1023.03	55.93	36.54
Raccoon	0.00	1644.95	123.13	20.43
Red fox	0.00	1943.33	89.28	23.18
Striped skunk	0.00	2421.42	109.38	26.33
White-tailed deer	0.00	4351.76	122.19	30.33

*For the all species combined results, the mean electrical current density was recorded for each species at each structure location. Species-specific means were then averaged together for an all species result at each structure and at each scale.

At the structure scale, we produced 5,912 maps of wildlife connectivity around individual structure locations for each species, totaling 47,296 structure scale maps across all eight species (example maps shown in S4 Fig). Structure-wide mean electrical current density was 361.55 ± 74.12 SE amps for all species combined and was highest for raccoon (123.13 ± 20.43 SE) and lowest for moose (55.93 ± 36.54 SE; Table 4).

The resulting electrical current maps predicting movement flow were a function of the two input layers: species occupancy probability and landscape resistance. Across all species and at each spatial scale of analysis, landscape resistance was low and species occupancy probability was high in natural landcover classes (forest, wetland, and shrub). Landscape resistance was generally high and species occupancy generally low in landcover influenced by anthropogenic use (road, agriculture, and development). Raccoon and skunk represented the exception, as these species had higher probability of occurrence in developed areas and lower levels of resistance to anthropogenic features, therefore showing higher levels of movement flow through areas of development. The composition of landscape variables greatly influenced electrical flow in the resulting Omniscape connectivity maps. Larger areas of natural forest cover (i.e., Green Mountain National Forest) typically had intermediate levels of electrical flow in the output maps: although there was higher electrical input into these areas from higher species occupancy probability, the electricity was able to ‘spread out’ in larger blocks of low resistance habitat, resulting in intermediate levels of flow. Similarly, smaller strips of natural landcover found in the higher-resistance agricultural and human development areas had high electrical flow, as electricity became constrained into smaller areas of ideal movement habitat.

### Objective 3: Roadway structure ranking

**Fig 3:**
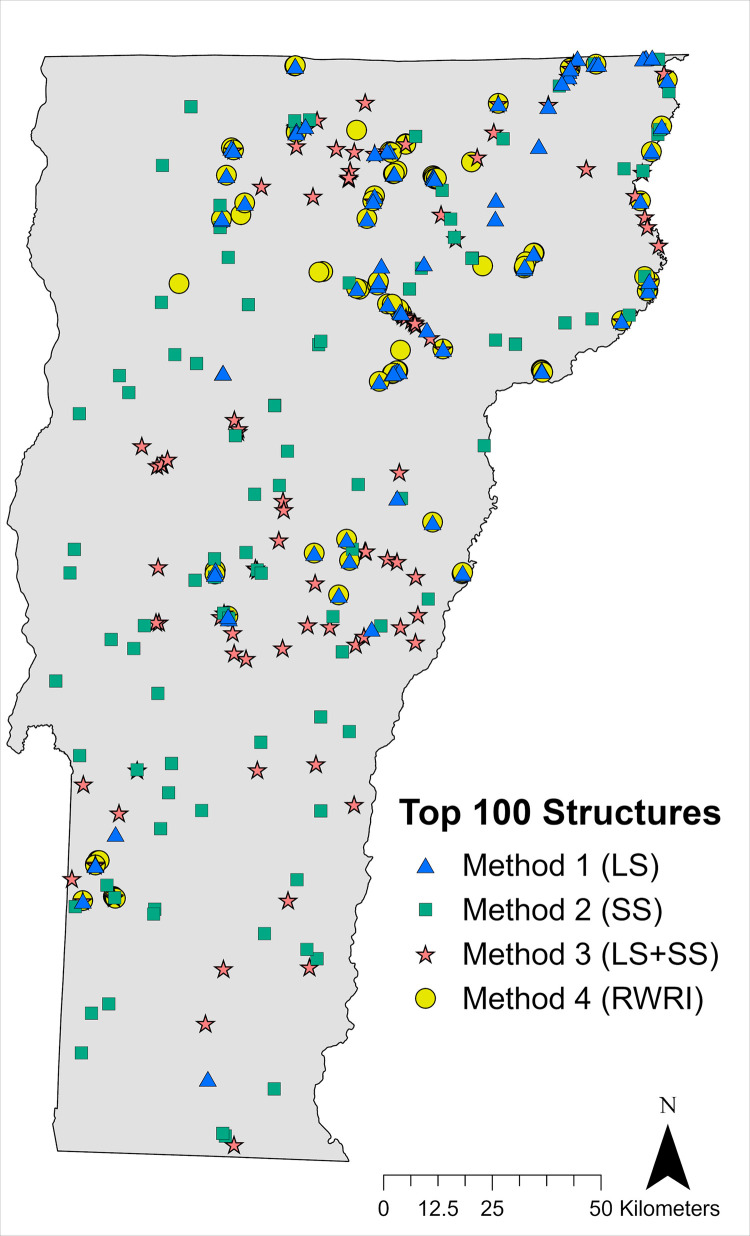
The top-ranking 100 roadway structures from four ranking methods. Structures were ranked from highest mean current density to lowest in each method. Method 1 considered landscape scale flow, Method 2 considered structure scale flow, Method 3 considered landscape and structure scale flow, and Method 4 used a Rarity-Weighted Richness Index (RWRI) to rank structures.

Spearman’s rank correlations showed a moderate to strong positive correlation between the rankings generated by the different methods (Table 5). Method 1 (landscape scale ranking) and 4 (landscape scale RWRI) had the strongest correlation (ρ = 0.94), as both methods ranked structures based on landscape scale current only. Methods 1 and 3 also showed a moderately positive correlation (ρ = 0.75), as did Methods 3 and 4 (ρ = 0.75). Methods 1 and 2 had a weaker positive correlation (ρ = 0.25), which was not unexpected since some structures may score high based on broader landscape-level movements of a species but lack adequate landcover for movement at the finer resolution within 50 m of the structure (Fig 3).

**Table 5:** Spearman’s rank correlations assessing the relationship between the four methods of transportation structure rankings. Percent of overlapping sites between ranking methods indicated in parentheses.

	Method 1	Method 2	Method 3	Method 4
**Method 1**	1.00			
**Method 2**	0.25 (4)	1.00		
**Method 3**	0.75 (16)	0.36 (3)	1.00	
**Method 4**	0.94 (69)	0.31 (3)	0.75 (15)	1.00

The Top 100 structures ranked by each method showed variable locations in the landscape as high-priority for wildlife-focused transportation investments (Fig 3). Overlap in sites between ranking methods was generally low and varied from 3 to 69 (mean = 18 ± 8.8 SE; Table 5). Method 1 highlighted structures in more natural areas of landcover, along the Green Mountain range and into the northeastern part of the state, as important for prioritization due to their location in areas of higher statewide movement flow for all species. Method 2 showed the greatest difference in structure locations from all ranking methods, as it only considered site-level characteristics within a small radius of structures and ignored statewide patterns of species movement flow. The Top 100 structures ranked with Method 2 were more well-distributed geographically and also found in areas lacking greater landscape scale current flow. Since Method 3 considered both LS and SS movement flow, the Top 100 structures for this method were still found in areas of higher statewide flow, but also in locations with good site-level characteristics; these structures were more geographically spread out than Method 1. Method 4, in contrast, selected the most geographically constrained Top 100 structures, concentrated in select areas containing high-density current for one or more species with rare current flow; these structures were found mostly in the northern part of the state.

### Evaluation of connectivity models

We detected the focal species 2,180 times across the 49 camera sites, which were selected in areas predicted to be ideal for wildlife movement and connectivity [65]. Species presence was observed at 15 sites for coyote, 33 for deer, 3 for moose, 13 for bear, 17 for bobcat, 18 for red fox, and 14 for skunk. Naïve probability of presence was 71.4% for carnivores, 67.3% for ungulates, and 81.6% for all species combined across all sites. Estimated connectivity scores for all species combined at the landscape scale and structure scale, standardized on a 0–100 scale, were 75.3 ± 7.9 SD and 72.1 ± 10.5 SD, respectively.

## Discussion

Our analysis demonstrates that multi-scale and multi-species omnidirectional connectivity models can be used to determine probable road crossing locations used by terrestrial mammal species, particularly in relation to existing roadway structures, to help guide transportation infrastructure mitigation efforts. Our connectivity modeling included a variety of species and results showed different predicted movement behaviors for different species, but performed well against an independent set of camera data at select roadway structure locations. Differences in model outputs reflect the variation in movement behavior, sensitivity to habitats, and species distributions. The flow of animal movement across the landscape was highest along the forested, mid-elevation foothills of the Green Mountains in central Vermont, especially for forest-obligate species. In contrast, the raccoon and skunk models showed more concentrated movements in developed areas, such as in urban centers of Burlington and Montpelier Vermont, whereas species such as bear and moose showed much lower levels of predicted movement in highly developed areas.

We developed connectivity models for each species as movement behaviors are species-specific, particularly in relation to roadways [6,35,67]. Species-specific maps allow managers and decision-makers to evaluate structures on a species-by-species basis, as needed, depending on management objectives. For example, in Vermont, decisions about retrofitting existing roadway structures with walking shelves or a solid dirt movement surface may be based on the value of a given structure for certain target species like bobcat or moose. We also combined each species map into an all-species map to provide a singular, more general depiction of connectivity for the entire group of focal species and for a broader structure ranking. This approach could be adjusted to meet different management objectives by applying weighted values to higher-priority species maps before combining results. For example, if transportation managers are primarily concerned with identifying high-priority deer crossing locations over other species, the deer-specific model could be multiplied by a weight before summing all species maps together. Focal species selected for this analysis were based on specific transportation and wildlife agency objectives to mitigate the impacts of roadways on larger terrestrial species that present higher risk to motorists (including bear, moose, and deer), as well as species that hold ecological, economic, and cultural value in the state [39]. While prioritizing infrastructure needs for these species may benefit multiple other taxa with similar movement and habitat needs, it would be beneficial for future work to include additional taxa with different or highly specific movement needs, such as amphibians and reptiles that require both upland and wetland habitat on either side of a roadway crossing, and aquatic organism passage through stream crossings. Previous work in the state resulted in the development of the Vermont Culvert Aquatic Organism Passage Screening Tool, to help identify priority stream crossing projects that would increase aquatic organism passage in the state [68].

Circuit-based connectivity models are widely used to predict areas of movement and functional connectivity for species [27,69]. Our approach integrated landscape resistance and species-specific patterns of occupancy. The inclusion of occupancy served to account for the distribution of species when quantifying connectivity, which may improve structure level assessments of connectivity over using a single input to serve as both the source of animals and landscape resistance. For example, a structure surrounded by high resistance movement habitat may still be more valuable for connectivity if species occupancy (and abundance) is very high near that site. However, a structure that is surrounded by low resistance movement habitat may be less valuable and experience less flow if the species does not occur near the site. Integrating both resistance and occurrence information may produce a more robust model that considers probable locations of a species as well as predicted responses to landscape variables during movements.

Connectivity analyses that incorporate multiple scales or resolutions generally provides more accurate depictions of species movement across a landscape [70]. However, connectivity studies often fail to consider the influence of multiple spatial scales on species responses to landcover [71]. Decreasing the grain size to a finer spatial resolution has also been shown to improve connectivity model performance [72]. In our approach, we integrated connectivity at two spatial scales to quantify and rank connectivity at existing roadway structures. Although it can be challenging to determine the best scales to use for connectivity analysis, we believe that these two scales adequately represented the group of species, fine-scale conditions at structures, and helped reduce biases associated with relying on a single spatial scale. For example, a high connectivity value at the structure scale for a given species could lead to the conclusion that the site is of high value to animal movement without considering the broader landscape scale. If the broader landscape scale reflected generally poor conditions for movement, then the high site level score is misleading. Incorporating the landscape scale information in this case essentially tempers the site level score and presumably results in a more accurate assessment of connectivity at the structure.

Similarly, connectivity modeling at the landscape scale was performed at the home range scale by using the average home range size for a given species as the moving window size. This allowed us to appropriately scale the extent of movement at any given point in the landscape to the typical ranging behavior of the species. However, it is important to recognize that road crossings driven by dispersal movements may occur outside of normal home range movement patterns. Depicting connectivity for dispersing animals could be accomplished by simply increasing the moving window size to an average dispersal distance for a given species [73].

Species-specific resistance surfaces could be improved by parameterizing values with empirical wildlife movement data, such as data from GPS-collared animals or genetic samples, though these data are not always available [74]. Furthermore, incorporating dynamism into circuit-based connectivity models is an emerging field of research that would contribute additional detail to wildlife movement predictions, since movement across roadways can vary depending on season, diel period, or other factors for some species [75,76]. Dynamic connectivity models may provide more detailed predictions of movement, however they can be challenging to build, as this requires gathering extensive data on species movement behaviors and landscape resistance associated with (sometimes rapidly) varying landscape conditions [75].

Quantifying connectivity at features or points of interest in the landscape, poses a challenge for any connectivity modeling analysis. We proposed four methods of ranking connectivity to provide a range of options that reflect different potential management objectives. Each method emphasized a particular objective, including to restore or promote landscape-level connectivity for a species (method 1), restore or promote fine-scale connectivity based on site-level characteristics (method 2), consider both landscape-level and fine-scale species movements for promoting connectivity (method 3), and prioritizing connectivity actions in areas where rarer species movements are represented (method 4). Each of the four ranking methods resulted in different assemblages and rankings of roadway structures, highlighting the need to clearly articulate the specific method used, the benefits, limitations, and assumptions of that method, and the broader objective of scoring structures or other features in a landscape. This is especially important as rankings often form the basis of decision-making around prioritizing investments in wildlife management. In the context of transportation management, rankings often identify structures or locations to prioritize for modifications to facilitate movement across road networks [77,78].

Circuit-based connectivity analysis clearly shows patterns of concentrated electrical (animal) flow and is useful for identifying important pinch-points and high-use movement paths in a landscape [79,80]. However, interpretation of circuit-based outputs can at times be misleading, especially for ‘low flow’ areas. For example, areas with low flow may represent truly poor areas of movement (e.g., an urban area for a forest dependent species), but may also represent areas that are highly conducive to flow, just not concentrated flow (e.g., a large forest block for the same species). This discrepancy creates challenges to interpretation. Classifying electrical current outputs into categories of impeded, diffuse, and intensified/channelized flow has been proposed to more clearly interpret patterns of flow and distinguish between poor low-flow areas (impeded) and good low-flow areas (diffuse) [33,81]. Our study focused on pinch points of concentrated electrical flow immediately near roadways, and within small analysis areas at specific roadway structures, therefore accounting for these low flow areas in larger habitat blocks was not as relevant to our objectives.

Circuit-based connectivity models are also difficult to validate; however empirical detection data from roadkill, telemetry, tracking, and wildlife camera traps have been used to evaluate performance in other studies [36,82,83,84]. Our evaluation of connectivity model results against available independent camera data suggested that predictions of increased connectivity coincided with higher naïve occupancy probabilities of ungulates and carnivores at these locations; however, cameras were only deployed at high-value sites with a limited sample size, and detection was not accounted for in occupancy estimates. These results support the connectivity results and provide some insight into the accuracy of the connectivity model predictions. Including data from sites characterized as low-value for connectivity may improve assessments of model performance, but these data were unavailable for this study. Additionally, the species-specific occurrence models used as model inputs performed well when tested against an independent empirical dataset of research-grade wildlife sightings reported through the iNaturalist community-science database [58]. Although the landscape resistance layer input was not tested against independent data, the validation of the occurrence models and congruence of camera data with areas of high predicted wildlife movement in resulting Omniscape models help support the model predictions and ultimately structure rankings.

Projections suggest that future development will threaten connectivity within Vermont and the broader region as trends show an increase in the construction of residential public roadways of 1,000 km per decade [85]. Transportation networks are wide-reaching, and prioritizing connectivity investments can be a difficult task for state transportation and wildlife management agencies that often work with limited funding to implement improvements [25]. The results and model inputs, such as species occurrence and landscape resistance values, can be applied to future scenarios of landscape change and roadway development to predict how species movements and connectivity may change under future landscape conditions, as was done in Pearman-Gillman et al. [86]. Quantifying landscape connectivity provides important information to develop effective management strategies that mitigate the impacts of transportation infrastructure on wildlife populations. Beyond transportation management, the results may be used to identify locations in Vermont for different conservation objectives, such as land protection or restoration of connectivity through fragmented areas. For example, the Vermont Legislature passed the Community Resilience and Biodiversity Act in 2023 that mandates protecting 30% of Vermont’s total land area by 2030 and 50% by 2050 and emphasizes the importance of connected lands for ecological functioning. The results provide maps that can be used to identify high-value sites for connectivity in support of state efforts to meet these protection goals. Similarly, results could be used to better quantify the connectivity value of wildlife corridors across the broader Northern Appalachians, such as those defined by the Staying Connected Initiative and other regional connectivity projects.

## Supporting information

S1 Fig Expert opinion survey used to gather information on species-specific landscape resistance values. Survey created using the LimeSurvey GmbH (2005) platform.
(PDF)

S2 Table Species occurrence models from Pearman-Gillman et al. (2020) used as source-strength inputs for the connectivity analysis. Top model parameter estimates shown with standard error and upper (UCI) and lower (LCI) confidence intervals. Models were developed as generalized linear mixed effect models that included random and fixed effects and explored the influence of 74 variables.
(PDF)

S3 Fig Averaging process used to buffer species-specific occurrence inputs into buffered areas lacking species occurrence data: New York, USA and Québec, Canada. (TIF)

S4 Fig Example of Omniscape analysis at the structure scale. The eight species-specific models run within a 100 m radius of transportation structure locations predicted different movement patterns and current densities around the structure. Resulting maps were clipped and mean current density was calculated within a 50 m radius of structures to minimize potential edge effects in the modeling.
(TIF)
