# Supplementary material for: Assessing the connectivity value of roadway structures for terrestrial mammals across the Northern Appalachian forest of Vermont
Source: PLoS One. 2025 Sep 4;20(9):e0331493. doi: 10.1371/journal.pone.0331493 (PMC12410740; doi:10.1371/journal.pone.0331493)
Supplement: S1 Fig — Survey created using the LimeSurvey GmbH (2005) platform. (PDF) [file pone.0331493.s001.pdf]

Vermont Wildlife Connectivity Survey

Your input from this survey will assist in the development of landscape resistance maps, to help model and map wildlife movement in the state of Vermont.

For complete information on the project and survey procedures, please review the Research Information Sheet at this link: Information Sheet

Welcome!

You are being requested to participate in this survey because you are a wildlife professional specializing in one or more terrestrial species in Vermont. Data collected in this survey will be incorporated into landscape resistance maps for each species, to help model species movements at two spatial scales.

Part I of this survey will ask you to think about the landscape-level movements of your species throughout the state of Vermont. Part II of the survey will ask you to think about the fine-scale movements of your species within a localized area. Part III of the survey is optional and allows you to write any additional comments.

This survey should be completed for a single species. If you have been asked to provide input on multiple species, please start a new survey submission for additional species.

This survey is anonymous.

The record of your survey responses does not contain any identifying information about you, unless a specific survey question explicitly asked for it.

If you used an identifying token to access this survey, please rest assured that this token will not be stored together with your responses. It is managed in a separate database and will only be updated to indicate whether you did (or did not) complete this survey. There is no way of matching identification tokens with survey responses.

Next

Focal Species

Please list the name of the species you are focusing on for this survey:

(Reminder: one species per survey)

Previous

Next

Part I: Landscape Scale Movement

Instructions:

The first part of this survey aims to understand the landscape-scale movements of wildlife species throughout the state of Vermont. The 17 land cover variables listed below are from the 2016 National Land Cover Database, and the road variables are from the Vermont Agency of Transportation. These data are at a 30-meter resolution, and will be used to model broader wildlife movement throughout the state.

Please review each variable and associated descriptions from the datasets in Column 1. Consider how individuals of your focal species would move through these landcover types in Vermont (with an emphasis on movement of the species, not solely the occurrence of the species).

In the second column, please assign a resistance value for each variable that represents how difficult it is for the species to make a movement through it. This resistance value may range from 1 (least difficult to move through) to 100 (most difficult), and 101 can be used to represent a complete barrier (impossible to move through). You may assign the same resistance value to variables that would have the same movement difficulty for the species.

See the project explanation video for additional information, or email cdrasher@uvm.edu with any questions.

Only numbers may be entered in these fields.

	Resistance Score (1-least resistant to 100-most resistant)
**Open Water**<br>NLCD 11: "All areas of open water, generally with less than 25% cover or vegetation or soil"	
**Developed: Open Space & Low-Medium Intensity**<br>NLCD 21: "Includes areas with a mixture of some constructed materials, but mostly vegetation in the form of lawn grasses. Impervious surfaces account for less than 20 percent of total cover. These areas most commonly include large-lot single-family housing units, parks, golf courses, and vegetation planted in developed settings for recreation, erosion control, or aesthetic purposes."<br>NLCD 22: "Includes areas with a mixture of constructed materials and vegetation. Impervious surfaces account for 20-49 percent of total cover. These areas most commonly include single-family housing units."<br>NLCD 23: "Includes areas with a mixture of constructed materials and vegetation. Impervious surfaces account for 50-79 percent of the total cover. These areas most commonly include single-family housing units."	
**Developed High Intensity**<br>NLCD 24: "Developed, High Intensity - Includes highly developed areas where people reside or work in high numbers. Examples include apartment complexes, row houses and commercial/industrial. Impervious surfaces account for 80 to 100 percent of the total cover."	
**Barren Land**<br>NLCD 31: "Barren areas of bedrock, desert pavement, scarps, talus, slides, volcanic material, glacial debris, sand dunes, strip mines, gravel pits and other accumulations of earthen material. Generally, vegetation accounts for less than 15% of total cover."	

Part II: Fine Scale Movement

Instructions:

The second part of this survey aims to understand the fine-scale movements of wildlife species adjacent to roads/transportation structures. The 14 land cover variables listed below are from the Vermont Center for Geographic Information, and the road variables are from the Vermont Agency of Transportation. These data are at a 0.5 meter resolution, and will be used to model wildlife movement in specific areas with a 100-meter radius.

Please review each variable and associated descriptions from the datasets in Column 1. Consider how individuals of your focal species would make fine-scale movements through these landcover types (with an emphasis on movement of the species, not solely occurrence of the species).

In the second column, please assign a resistance value for each variable that represents how difficult it is for the species to make a movement through it. This resistance value may range from 1 (least difficult to move through) to 100 (most difficult), and 101 can be used to represent a complete barrier (impossible to move through). You may assign the same resistance value to variables that would have the same movement difficulty for the species.

See the project explanation video for additional information, or email cdrasher@uvm.edu with any questions.

Variables here are self-descriptive. For more information on how these variables were derived, view the VCGI report here: https://vcgi.nyc3.digitaloceanspaces.com/portal-pages/VT-Land-Cover-2016-Final-Report-v3.pdf

Only numbers may be entered in these fields.

	Resistance Score (1-least resistant to 100-most resistant, 101-impenetrable)
**Canopy Cover**	
**Water**	
**Grass/shrub**	
**Agriculture**	
**Wetland**	
**Bare Soil**	
**Other Paved**	
**Railroad**	
**High-traffic Town Roads**<br>Class I & II Town Highways.	
**Moderate-traffic Town Roads**<br>Class III Town Highways.	
**Low-traffic Town Roads**<br>Class IV Town Highways.	
**Low/No Traffic Roads**<br>Private Roads, Discontinued Highways, All Other Roads, Legal Trails, Unconfirmed Legal Trails, National or State Forest Highways.	
**State Road System**<br>State Highways, US Highways, US Highway Ramps, US Divided Highways.	
**Interstate System**<br>Interstate Highways, Interstate Highway Ramps.	

Additional comments

Please list any additional comments below:

Previous

Submit
