# Supplementary material for: Assessing the connectivity value of roadway structures for terrestrial mammals across the Northern Appalachian forest of Vermont
Source: PLoS One. 2025 Sep 4;20(9):e0331493. doi: 10.1371/journal.pone.0331493 (PMC12410740; doi:10.1371/journal.pone.0331493)
Supplement: S2 Table — Top model parameter estimates shown with standard error and upper (UCI) and lower (LCI) confidence intervals. Models were developed as generalized linear mixed effect models that included random and fixed effects and explored the influence of 74 variables. (PDF) [file pone.0331493.s002.pdf]

Species	Model, Covariates	β Estimate	SE	LCI	UCI
Black bear	Mean ~ prop_mature_forest + prop_all_roads + prop_forest_5k + mean_annual_precip_mm_5k + prop_fagugran_5k þ (1 | State) + (1 | Expert) + (1 | Site)				
	(Intercept)	25.64	11.34	3.42	47.86
	prop_mature_forest	3.27	0.86	1.59	4.95
	prop_all_roads	-12.47	2.15	-16.68	-8.26
	prop_forest_5k	6.16	0.88	4.43	7.90
	mean_annual_precip_mm_5k	-21.90	8.50	-38.57	-5.24
	prop_fagugran_5k	2.40	1.01	0.42	4.38
Bobcat	Mean ~ prop_developed + prop_forest_edge + prop_agriculture + (1 | Expert) + (1 | Site)				
	(Intercept)	0.22	0.36	-0.48	0.93
	prop_developed	-2.6	0.50	-3.58	-1.62
	prop_forest_edge	1.02	0.42	0.19	1.85
	prop_agriculture	1.42	0.52	0.40	2.44
Coyote	Mean ~ prop_waterbodies + prop_forest_edge + prop_major_roads_3k + prop_wetland_3k + prop_agriculture + (1 | Expert) + (1 | Site)				
	(Intercept)	1.42	0.72	0.01	2.82
	prop_waterbodies	-4.08	0.97	-5.99	-2.18
	prop_forest_edge	2.79	0.54	1.73	3.86
	prop_major_roads_3k	-32.05	9.94	-51.54	-12.56
	prop_wetland_3k	2.85	1.34	0.21	5.48
	prop_agriculture	1.31	0.71	-0.07	2.70
Moose	Mean ~ prop_young_forest + prop_developed + prop_shrubland + mean_fall_tmax_degC + prop_forest_5k + (1 | Expert) + (1 | Site)				
	(Intercept)	8.13	1.61	4.97	11.29
	prop_young_forest	7.02	2.93	1.27	12.76
	prop_developed	-4.59	0.78	-6.11	-3.06
	prop_shrubland	5.11	1.37	2.43	7.79
	mean_fall_tmax_degC	-73.71	8.98	-91.32	-56.1
	prop_forest_5k	3.52	0.65	2.25	4.79
Raccoon	Mean ~ prop_agriculture_500m + prop_mature_forest_500m + mean_DEM_km_500m + prop_oak_500m + prop_developed_500m + (1 | Expert) + (1 | Site)				
	(Intercept)	1.65	0.71	0.27	3.04
	prop_agriculture_500m	3.04	0.75	1.58	4.51
	prop_mature_forest_500m	1.21	0.54	0.15	2.27

Species	Model, Covariates	β Estimate	SE	LCI	UCI
	mean_DEM_km_500m	-2.09	0.66	-3.37	-0.80
	prop_oak_500m	1.66	0.83	0.03	3.3
	prop_developed_500m	2.26	0.60	1.07	3.44
Red fox	Mean ~ prop_agriculture + prop_high_dev + mean_winter_precip_mm_3k + prop_shrubland_3k + (1 | Expert) + (1 | Site)				
	(Intercept)	-3.16	1.77	-6.63	0.3
	prop_agriculture	3.28	0.61	2.09	4.47
	prop_high_dev	-3.23	1.21	-5.60	-0.86
	mean_winter_precip_mm_3k	12.65	6.30	0.31	24.99
	prop_shrubland_3k	3.5	2.10	-0.63	7.62
Striped skunk	Mean ~mean_DEM_km_500m + prop_mature_forest_500m + prop_agriculture_500m + prop_forest_edge_500m + (1 | Expert) + (1 | Site)				
	(Intercept)	1.91	0.79	0.36	3.45
	mean_DEM_km_500m	-6.25	0.60	-7.44	-5.07
	prop_mature_forest_500m	0.91	0.58	-0.23	2.06
	prop_agriculture_500m	3.40	0.76	1.91	4.88
	prop_forest_edge_500m	0.74	0.49	-0.22	1.70
White-tailed deer	Mean ~ prop_agriculture + prop_high_dev + prop_mature_forest + prop_hemlock_tamarack_cedar_3k + (1 | EcoRegion) + (1 | Expert) + (1 | Site)				
	(Intercept)	1.17	0.68	-0.17	2.5
	prop_agriculture	4.22	0.83	2.60	5.84
	prop_high_dev	-10.52	0.84	-12.17	-8.88
	prop_mature_forest	1.47	0.62	0.27	2.68
	prop_hemlock_tamarack_cedar_3k	10.5	1.69	7.18	13.82
