# Supplementary figures and images for: Assessing the connectivity value of roadway structures for terrestrial mammals across the Northern Appalachian forest of Vermont
Source: PLoS One. 2025 Sep 4;20(9):e0331493. doi: 10.1371/journal.pone.0331493 (PMC12410740; doi:10.1371/journal.pone.0331493)

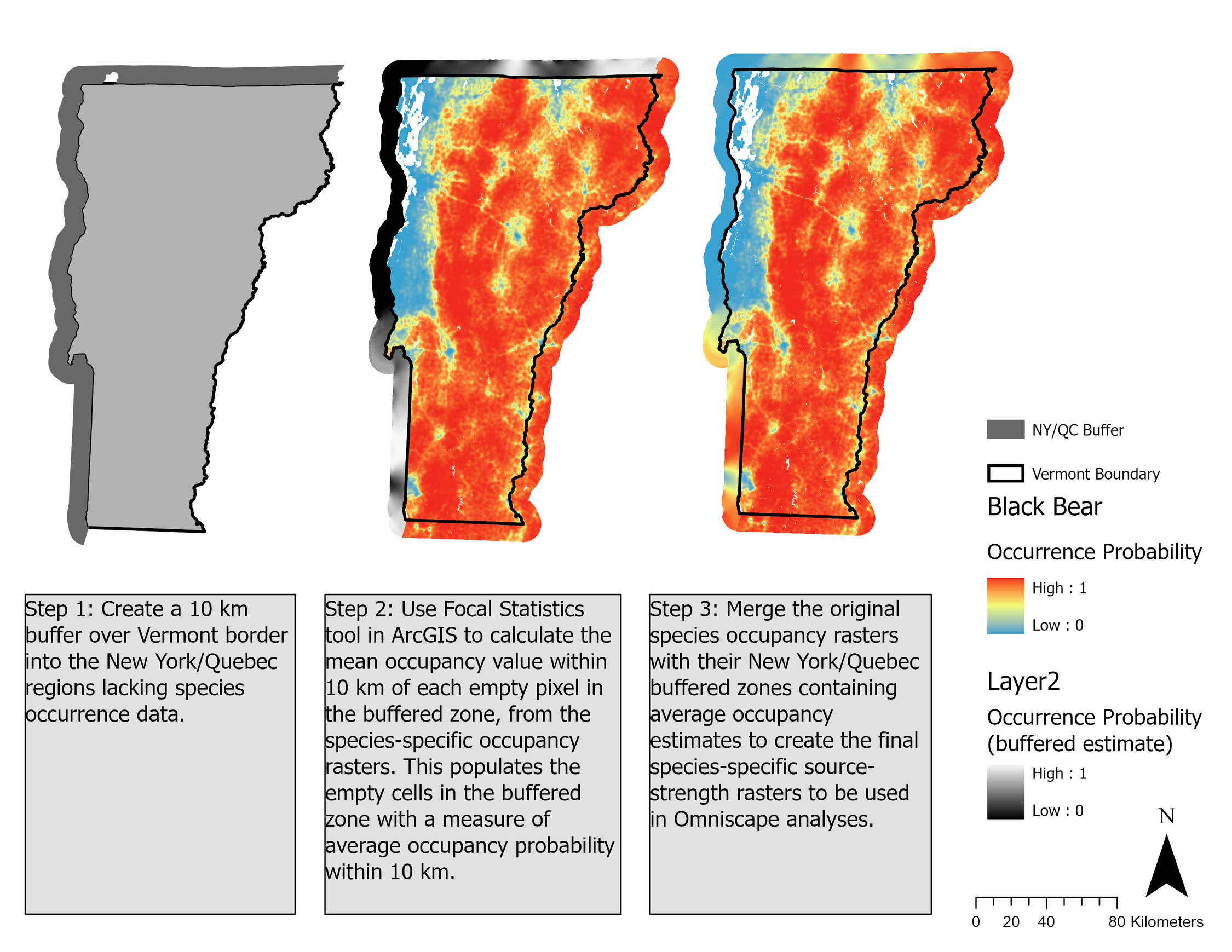

Supplement: S3 Fig — (TIF) [file pone.0331493.s003.tif]

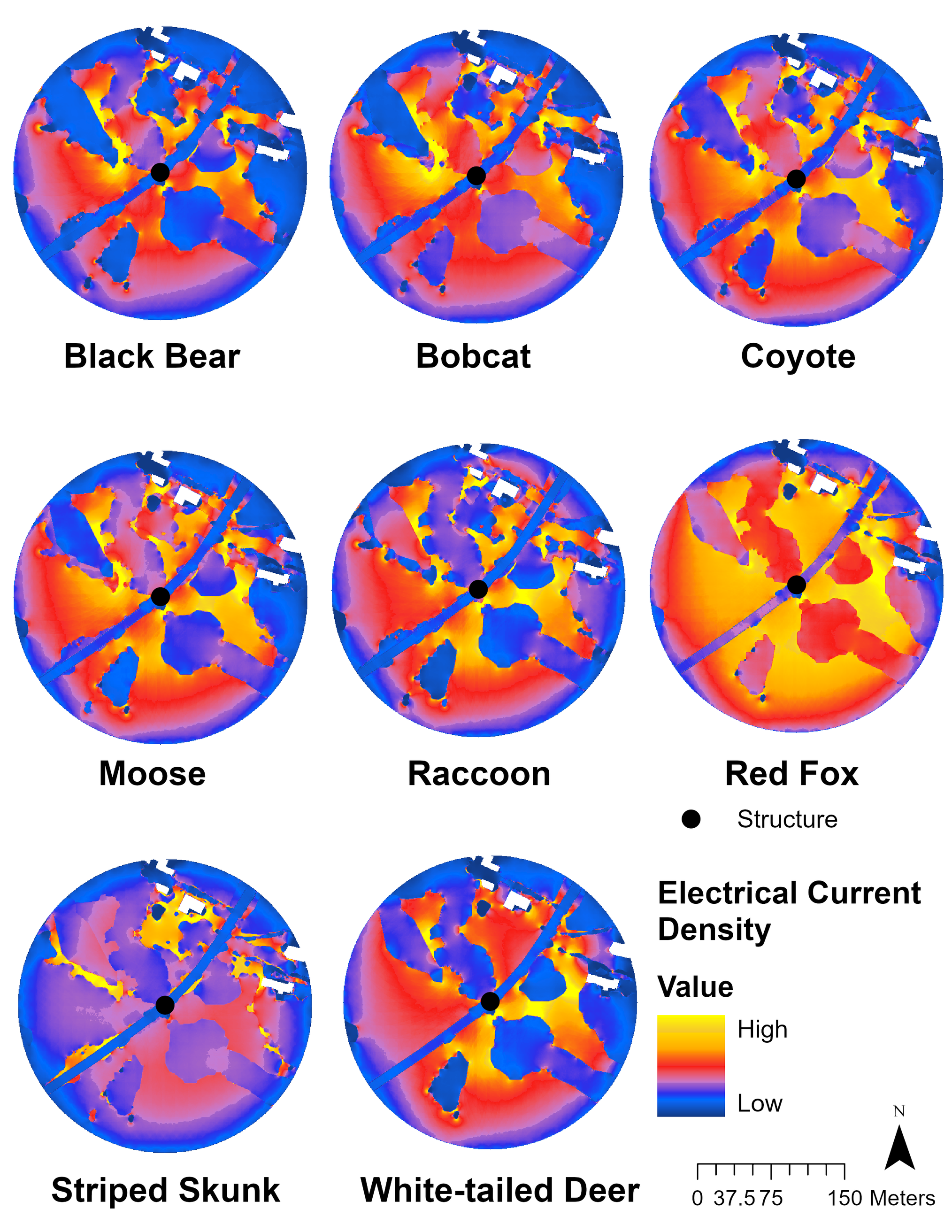

Supplement: S4 Fig — The eight species-specific models run within a 100 m radius of transportation structure locations predicted different movement patterns and current densities around the structure. Resulting maps were clipped and mean current density was calculated within a 50 m radius of structures to minimize potential edge effects in the modeling. (TIF) [file pone.0331493.s004.tif]
